# Prognostic signature and immune efficacy of m^1^A‐, m^5^C‐ and m^6^A‐related regulators in cutaneous melanoma

**DOI:** 10.1111/jcmm.16800

**Published:** 2021-07-21

**Authors:** Xian rui Wu, Zheng Chen, Yang Liu, Zi zi Chen, Fengjie Tang, Zhi zhao Chen, Jing jing Li, Jun lin Liao, Ke Cao, Xiang Chen, Jianda Zhou

**Affiliations:** ^1^ Department of Plastic Surgery of Third Xiangya Hospital Central South University Changsha Hunan China; ^2^ Department of Plastic Surgery of Third Xiangya Hospital Central South University Changsha Hunan China; ^3^ Department of Plastic Surgery of Xiangya Hospital Central South University Changsha Hunan China; ^4^ Departments of Medical Cosmetology The First Affiliated Hospital University of South China Hengyang Hunan China; ^5^ Department of Oncology of Third Xiangya Hospital Central South University Changsha Hunan China; ^6^ Department of Dermatology The Xiangya Hospital Central South University Changsha Hunan China

**Keywords:** cutaneous melanoma, immunotherapy, m1A, m5C, m6A, prognosis, tumour microenvironment

## Abstract

Cutaneous melanoma (CM) is an aggressive cancer; given that initial and specific signs are lacking, diagnosis is often late and the prognosis is poor. RNA modification has been widely studied in tumour progression. Nevertheless, little progress has been made in the signature of N^1^‐methyladenosine (m^1^A), 5‐methylcytosine (m^5^C), N^6^‐methyladenosine (m^6^A)‐related regulators and the tumour microenvironment (TME) cell infiltration in CM. Our study identified the characteristics of m^1^A‐, m^5^C‐ and m^6^A‐related regulators based on 468 CM samples from the public database. Using univariate, multivariate and LASSO Cox regression analysis, a risk model of regulators was established and validated by a nomogram on independent prognostic factors. The gene set variation analysis (GSVA) and the Kyoto Encyclopedia of Genes and Genomes (KEGG) clarified the involved functional pathways. A combined single‐sample gene set enrichment analysis (ssGSEA) and CIBERSORT approach revealed TME of regulator‐related prognostic signature. The nine‐gene signature stratified the patients into distinct risk subgroups for personalized prognostic assessment. Additionally, functional enrichment, immune infiltration and immunotherapy response analysis indicated that the high‐risk group was correlated with T‐cell suppression, while the low‐risk group was more sensitive to immunotherapy. The findings presented here contribute to our understanding of the TME molecular heterogeneity in CM. Nine m^1^A‐, m^5^C‐ and m^6^A‐related regulators may also be promising biomarkers for future research.

## 1 INTRODUCTION

Melanoma is an aggressive form of skin cancer. Due to genetically complex development of cutaneous melanoma (CM), its therapeutic management remains challenging worldwide.^1^ Despite the various aggressive intervention, patients continue to have a high recurrence rate, which is a poor prognostic factor for CM.^2^ Thus, there is an urgent need to develop more appropriate and effective prognostic biomarkers of CM. Gene expression has recently emerged as a promising prognostic factor for various cancers. Thus, it is worth looking for novel and appropriate molecular biomarkers to unveil the mechanistic biological processes in treatment‐resistant CM.

RNA modification is a way of post‐transcriptional regulation, which works as an additional link between transcription and translation and is crucial for the event of many diseases. Over one hundred styles of RNA modifications have been discovered, the most common one being m^6^A methylation.^3^ m^5^C and m^1^A are new RNA modifications that have attracted widespread attention in recent years.^4^ RNA modifications, involving m^1^A, m^5^C and m^6^A, are implicated in regulating cancer cell proliferation, transformation, invasion and different malignant behaviours. The m^6^A‐related regulators as prognostic biomarkers have reported in many research studies. These regulators were also mentioned as playing a key role in multiple processes of tumorigenesis and progression.^5^ YTHDF1 may act as an m^6^A‐related regulators in colorectal cancer to promote the malignant phenotypes through inhibiting the Wnt/ß‐catenin pathway.^6^ NSUN2 is an m^5^C‐regulatory gene correlates with lower survival rate in patients with gastrointestinal (GI) cancer by regulating RNA methylation modification via the ErbB signalling pathway.^7^ Similar to m^6^A and m^5^C, TRMT6 has been found to mediate the MYC, and also the PI3K/Akt signalling pathway in vitro, thereby downregulating m^1^A and affecting hepatocellular carcinoma (HCC) prognosis.^8^ Collectively, the underlying correspondence between m^1^A‐, m^5^C‐ and m^6^A‐related regulators and varied tumours sparked a revived interest in developing original prognostic biomarkers. Consequently, the signature of m^1^A‐, m^5^C‐ and m^6^A‐related regulators in CM is worth further investigations.

Immunotherapy showed superb clinical effectiveness in a minority of CM patients with long‐lasting effects.^9^, ^10^ Yet, the vast majority of patients have to endure the cost of frequent and serious immune‐related adverse events, which greatly hampers the actual efficacy.^11^ It has been widely accepted that tumour microenvironment (TME) plays a critical role in malignancy evolvement and immune regulation. TME incorporates not solely tumour cells but also stromal cells (fibroblasts and macrophages), and immune cell infiltration (ICI), chemokines and growth factors. Alternative TME components directly or indirectly interact with tumour cells to cause changes in a variety of physical behaviours, such as apoptosis resistance,^12^ hypoxia tolerance^12^ and immune dysfunction.^13^ With the deepening of the understanding of the TME, several studies have demonstrated that TME plays a pivotal role in tumour progression, immune escape and immunotherapy response.^14^ Therefore, an extensive analysis of the TME landscape can effectively improve the ability to guide and predict the immunotherapy response.

This study set out to identify the potential signature of m^1^A‐, m^5^C‐ and m^6^A‐related regulators to improve prognostic evaluation of CM. We built a novel nine‐regulator (including *UNG*, *FMR1*, *MBD4*, *MBD2*, *NEIL1*, *WTAP*, *UHRF2*, *YTHDF1* and *RBM15B*) signature on the TCGA database. By integrating univariate, multivariate and LASSO Cox regression analyses, we established a regulator‐related risk predictive model to distinguish the level of risk among patients with CM. The importance and originality of this study are that it further revealed the underlying connection between the regulator‐related risk predictive signature and the ICI characteristics of TME. This novel signature could be used to evaluate the sensitivity of CM patients to immunotherapy.

## 2 MATERIALS AND METHODS

### 2.1 Selection of m^1^A‐, m^5^C‐ and m^6^A‐related regulators

A total of 48 m^1^A‐, m^5^C‐ and m^6^A‐related regulators were collected from previously published studies. According to the available data, *METTL3*, *METTL14*, *RBM15*, *RBM15B*, *WTAP*, *KIAA1429*, *CBLL1*, *ZC3H13*, *ALKBH5*, *FTO*, *YTHDC1*, *YTHDC2*, *YTHDF1*, *YTHDF2*, *YTHDF3*, *IGF2BP1*, *HNRNPA2B1*, *HNRNPC*, *FMR1*, *LRPPRC* and *ELAVL* are m^6^A‐related regulators^15^, ^16^; *DNMT1*, *DNMT3A*, *DNMT3B*, *MBD1*, *MBD2*, *MBD3*, *MBD4*, *MECP2*, *NEIL1*, *NTHL1*, *SMUG1*, *TDG*, *UHRF1*, *UHRF2*, *UNG*, *ZBTB33*, *ZBTB38*, *ZBTB4*, *TET1*, *TET2* and *TET3* are m^5^C‐related regulators^17^; and *TRMT10C*, *TRMT61B*, *TRMT6*, *TRMT61A*, *ALKBH3*, *ALKBH1*, *YTHDC1*, *YTHDF1*, *YTHDF2* and *YTHDF3* are m^1^A‐related regulators.^18^

### 2.2 Data Acquisition

All of the clinical patient data, mutations, copy‐number variation (CNV) and mRNA expression data were downloaded from the TCGA (http://gdc.cancer.gov). Patients from the TCGA‐SKCM (*N* = 468) were enrolled to form the internal training set. Furthermore, the GSE100797 data set (*N* = 25) was obtained from the Gene Expression Omnibus (GEO, http://www.ncbi.nlm.nih.gov/geo) as an external validation set to better validate the prognostic predictive power of the prognostic gene signature. Eligible subjects met the following selection criteria: (1) complete clinical information available and (2) survival time more than 90 days.

### 2.3 Establishment and validation for the prognostic signature of m^1^A‐, m^5^C‐ and m^6^A‐related regulators

A total of 46 regulators expressed in TCGA‐SKCM were enrolled in the survival analysis (Figure S5). The identification of m^1^A‐, m^5^C‐ and m^6^A‐related prognostic genes was carried out using univariate Cox regression analysis, and genes were considered significant with a cut‐off of *p* < 0.05. The selected factors in the LASSO regression were analysed by multivariate analysis. The risk score was generated as follows:

$$\text{risk score =}\;\frac{e^{\text{sum}(\text{each gene's expression levels}\;\times \;\text{corresponding coefficient)}}}{e^{\text{sum}(\text{each gene's mean expression levels}\;\times \;\text{corresponding coefficient)}}}.$$

The patients were stratified into high‐risk and low‐risk groups based on the median risk score. For the evaluation of the overall survival (OS) of high‐ and low‐risk groups, the Kaplan‐Meier (K‐M) survival analysis was performed by the R package ‘survival’. The same analysis was also conducted in the external validation set. Clinical information for the training set and the external validation set is presented in Table 1 and Table S5, respectively. The assessment of risk score prognostic efficiency was conducted based on the areas under the curve (AUCs) of the time‐dependent receiver operating characteristic (ROC) curve in the R package ‘TimeROC’.^19^

**TABLE 1:** Different clinicopathological features of the regulator‐related risk subgroups in TCGA‐SKCM

Clinical variables	Total (*N* = 342)	Risk group		*P*‐value
		High (*n* = 171)	Low (*n *= 171)	
Gender				1
Female	129 (37.7%)	64 (37.4%)	65 (38.0%)	
Male	213 (62.3%)	107 (62.6%)	106 (62.0%)	
Age				0.013
<60	180 (52.6%)	78 (45.6%)	102 (59.6%)	
≥60	162 (47.4%)	93 (54.4%)	69 (40.4%)	
Stage				0.913
High stage	154 (45.0%)	76 (44.4%)	78 (45.6%)	
Low stage	188 (55.0%)	95 (55.6%)	93 (54.4%)	
T				0.034
T_1_‐T_2_	128 (37.4%)	54 (31.6%)	74 (43.3%)	
T_3_‐T_4_	214 (62.6%)	117 (68.4%)	97 (56.7%)	
N				0.54
N_1_‐N_2_	303 (88.6%)	151 (88.3%)	152 (88.9%)	
N_3_‐N_4_	39 (11.4%)	20 (11.7%)	19 (11.1%)	
M				1
M_0_	331 (96.8%)	167 (97.7%)	164 (95.9%)	
M_1_	11 (3.2%)	4 (2.3%)	7 (4.1%)	
Radiotherapy				0.132
No	232 (67.8%)	123 (71.9%)	109 (63.7%)	
Yes	110 (32.2%)	48 (28.1%)	62 (36.3%)	
Chemotherapy				0.374
No	211 (61.7%)	110 (64.3%)	101 (59.1%)	
Yes	131 (38.3%)	61 (35.7%)	70 (40.9%)	

### 2.4 Independent prognostic roles of the regulator‐related signature

The univariate and multivariate Cox regression analyses were performed to test the hypothesis that the prognostic gene signature could be independent of other clinical parameters (including gender, radiotherapy, chemotherapy, N stage, T stage, M stage, stage and age). *P* < 0.05 was considered to be statistically significant.

### 2.5 Development of a nomogram

A nomogram was constructed based on the independent prognostic factors by the survival and the rms R package. The calibration curves and the concordance index (C‐index), ranging from 0.5 to 1.0, were used to gauge the model's ability to predict prognosis. The value of 0.5 and 1.0 represents a random chance and the optimal performance with the prognosis model, respectively.

### 2.6 GSVA and functional annotation

The ‘GSVA’ R package was used to test the enrichment of m^1^A‐, m^5^C‐ and m^6^A‐related regulatory gene signatures in the normalized gene expression table. Non‐parametric tests and unsupervised method were bound to compare the number of the pathway and biological process activity in the samples of an expression data set.^20^ Adjusted *P* with a value less than 0.05 was considered statistically significant.

### 2.7 Pathway analysis for the differentially expressed genes (DEGs) of the regulator‐related risk model

The DEGs between different risk groups were analysed with function of the Limma version 3.36.2 R package.^21^ DEGs with an absolute log_2_ fold change >1 and an adjusted *p* < 0.05 were included in the subsequent analysis. KEGG database is one of the most widely used techniques for determining the signalling pathways of DEGs. The calculation was completed with the clusterProfiler package in R software, and statistical significance was established at 0.05 level.

### 2.8 Estimation of TME immune cell infiltration

We utilized the ssGSEA algorithm to quantify the relative abundance of each cell infiltration in the cutaneous melanoma TME. The enrichment score calculated by the ssGSEA was utilized to represent the relative abundance of each TME‐infiltrating cell in each sample. The gene set for marking each TME infiltration immune cell type was stored in various human immune cell subtypes, including activated CD8 T cells, activated dendritic cells, macrophages, natural killer T cells and regulatory T cells.^14^

### 2.9 ESTIMATE algorithm

ESTIMATE is a well‐established algorithmic tool in the prediction of tumour purity, with ESTIMATE score generated by 141 immune genes and 141 stromal gene expression profiles, using the ESTIMATE R package. Five rounds of gene filtering distinguished the different signatures of m^1^A‐, m^5^C‐ and m^6^A‐related regulators: i) a ‘stromal signature’ for the stroma and ii) an ‘immune signature’ for the ICI in tumour tissue. Statistical significance was calculated by integrating the difference between the empirical cumulative distribution function, which is similar to the one used in GSEA, but instead based on absolute expression rather than differential expression.

#### 2.9.1 Statistical analysis

All analyses were carried out by the R software (version 3.5.2). Distributions of OS were compared using the log‐rank test. The C‐index was used to assess the probability that a prognostic signature with a high value could reflect poor survival. *P* < 0.05 was considered statistically significant. For multiple comparisons, the Bonferroni corrections were applied following the analysis of variance, and *p* < 0.05/number of tests served as the significance threshold.

## 3 RESULTS

### 3.1 The genetic landscape and expression levels of m^1^A‐, m^5^C‐ and m^6^A‐related regulators

**FIGURE 1:**
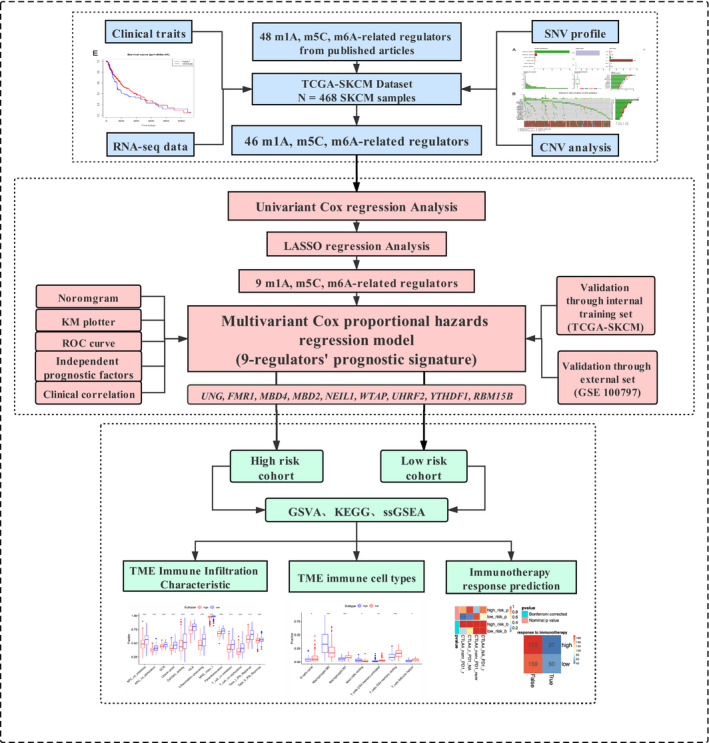
Workflow diagram of the present study

**FIGURE 2:**
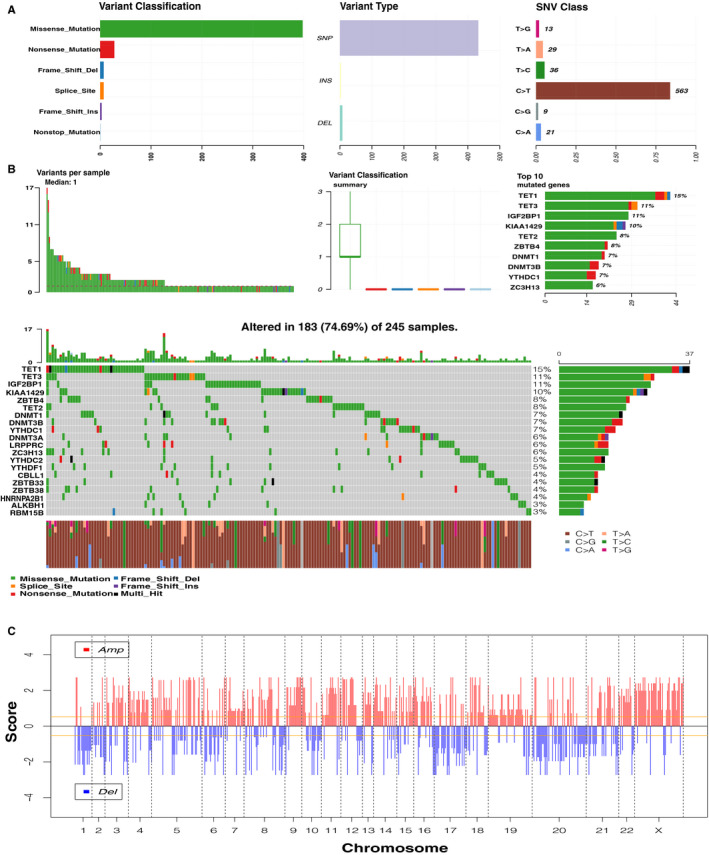
Genetic landscape of 46 m^1^A‐, m^5^C‐ and m^6^A‐related regulators. (A) The overview of mutation profiling in the 46 regulators from the TCGA‐SKCM data sets. (B) The mutation frequency of the 46 regulators in 245 samples (74.69%). The panel in the middle contains the specific mutation context of the top 20 regulators. Each mutation frequency on the right number; each column represented one sample; right bar chart presents variant‐type proportion; TMB distribution on the upper histogram. (C) Genomic visualization of CNV patterns in the 46 regulators. TMB: tumour mutation burden. TCGA‐SKCM: The Cancer Genome Atlas Skin Cutaneous Melanoma. OS: overall survival

**FIGURE 3:**
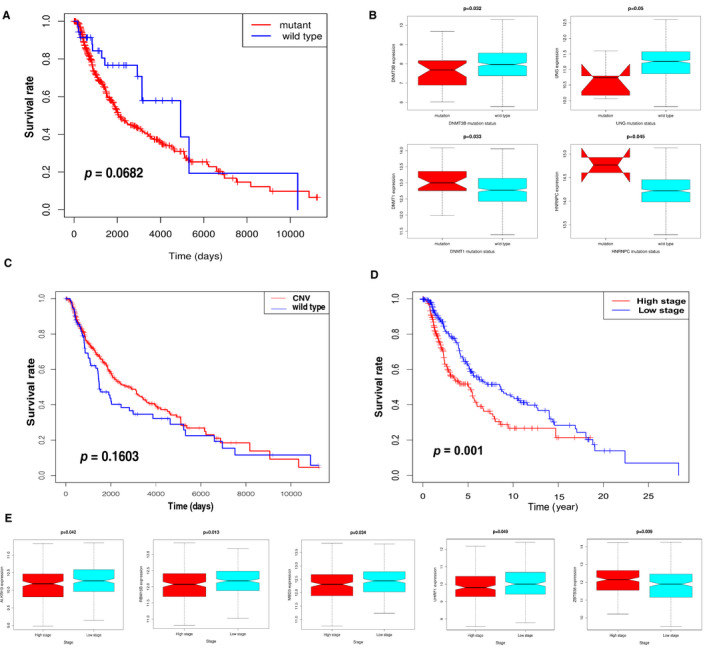
Relationship between the expression levels of m^1^A‐, m^5^C‐ and m^6^A‐related regulators and clinical features. (A) Survival analysis for SNV‐mediated regulators in TCGA‐SKCM patients. (B) Expression for four regulators with different mutation status. (*p* < 0.05). (C) The Kaplan‐Meier analysis of the 46 regulators with CNV in TCGA‐SKCM samples. (D) The Kaplan‐Meier analysis for high‐ and low‐stage groups according to 46 regulators. Low stage (blue curve): clinical TNM 1,2 stages; high stage (red curve): clinical TNM 3,4 stages. (E) The statistically significant difference in the expression of five regulators at different clinical stages (*p* < 0.05)

Figure 1 depicts the workflow diagram of the present study. A total of 46 m^1^A‐, m^5^C‐ and m^6^A‐related regulators were ultimately selected to perform the following analysis (Table S1). The incidence of SNV and CNV of 46 regulators was summarized in TCGA skin cutaneous melanoma (SKCM). Figure 2 presents the landscape of alteration obtained from the preliminary analysis of the 46 regulators. Missense mutation was the most frequent mutation event (Figure 2A). The top 20 mutated genes were identified based on the number of mutations in TCGA‐SKCM using the ‘maftools’ R package (Figure 2B). Among 245 samples, 183 experienced mutations of regulators, with a frequency of 74.69%. It was found that the *TET1* exhibited the highest mutation frequency. The investigation of the 46 regulators exhibited a prevalent CNV alteration and amplification in copy number. Figure 2C displays the CNV alteration location of the 46 regulators. A total of 467 CM samples were included for SNV studies. For CNV analysis, there were 416 CM samples. Survival analysis revealed that CM with SNV had a worse prognosis than that without mutations (Figure 3A). In contrast, there were no significant differences between CNV and wild type (Figure 3C). Four of 44 regulators were identified in 468 CM samples that had a significant association with different SNV patterns (Table S2). *DNMT3B* and *UNG* with SNV were linked to reduced mRNA expression, while increases were observed for *DNMT1* and *HNRNPC* (Figure 3B). Dose compensation effects were a contributing factor for the relationship between CNV alterations and gene expression levels.^22^ The next analysis focused on how CNV patterns attributed to expression of regulators. The correlation between the two in 416 CM samples is interesting because increased copy numbers of 45 regulators showed high expression, while deletions presented low expression (Figure S1). Only *IGF2BP* lacked a meaningful result (Table S2). These findings indicated that SNV and CNV of m^1^A‐, m^5^C‐ and m^6^A‐related genes could affect the expression of key regulatory molecules and contribute to CM progression.

### 3.2 Identification of the regulatory gene expression relevant to clinical prognosis

To explore the clinical signature of m^1^A‐, m^5^C‐ and m^6^A‐related regulators in CM, the K‐M survival analysis was implemented to evaluate the relationship between clinicopathological features and regulators using the data from TCGA‐SKCM. TNM stages 1 and 2 were defined as low stage, whereas TNM stages 3 and 4 were marked as high stage. The survival analysis displayed that the high stage had poorer outcomes in SKCM patients (Figure 3D). The heat map of 46 regulators’ expression was clustered at different stages (Figure S2); although the majority of the regulators showed no significant differences in the expression levels between the high stage and the low stage, we found a significant difference in the expression of five regulators. Figure 3E provides a box plot diagram of the expression of four regulators (*ALKBH3*, *RBM15B*, *MBD3* and *UHRF1*) that negatively correlated with clinical TNM stages. In contrast, *ZBTB38* positively correlated with clinical TNM stages. Hence, m^1^A‐, m^5^C‐ and m^6^A‐related regulators were related not only to the clinical TNM stage but also to the prognosis of CM patients. Collectively, m^1^A‐, m^5^C‐ and m^6^A‐related regulatory gene expression levels substantially correlated with the clinical TNM stage and prognosis in CM.

### 3.3 Construction of regulator‐related prognostic risk model

**FIGURE 4:**
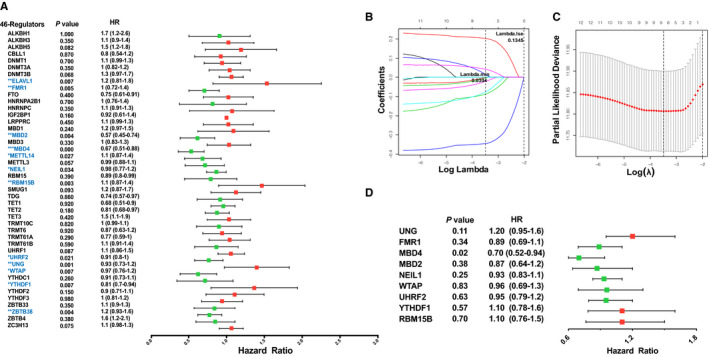
Construction of m^1^A‐, m^5^C‐ and m^6^A‐related regulatory gene prognostic signature in TCGA‐SKCM training set. (A) Forest plot of the univariate Cox regression analysis for the 46 regulators. Identification of 12 significant regulators. (*p < 0.05, **p < 0.01 and ***p < 0.001). (B, C) LASSO coefficient profiles of the 12 regulators. Cross‐validation for tuning parameter selection in the LASSO model. (D) Forest plot for the nine regulators with prognostic value in the multivariate Cox regression model

**FIGURE 5:**
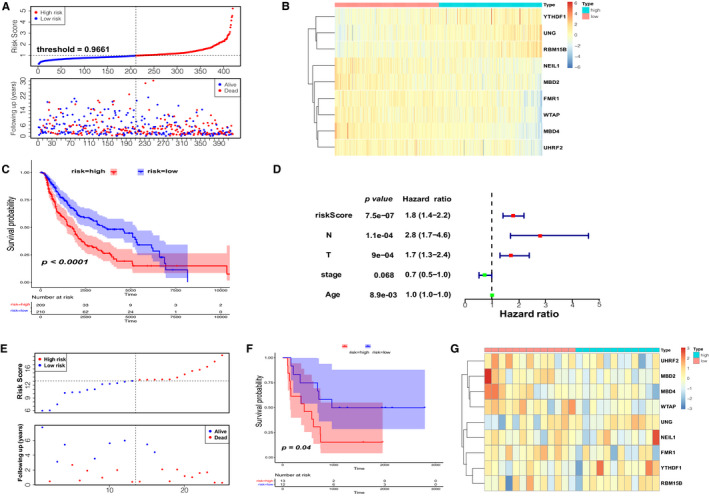
Prognostic signature of the nine m^1^A‐, m^5^C‐ and m^6^A‐related regulators in internal and external data set. (A) The distributions of prognostic signature‐based risk scores. (B) The heat map of the expression of the nine regulators in different risk subgroups. (C) The K‐M analysis showed that patients in the low‐risk group presented better OS than the high‐risk group. This analysis was based on the survival information of samples in the training set. The red line represents the high‐risk cluster, whereas the blue line indicates the low‐risk cluster. (D) Forrest plot of the independent prognostic factors in CM. (E) The distributions of risk score and survival status in the external validation set (GSE100797). (F) K‐M prognosis curve of the validation set. (G) The heat map of regulators’ expression clustered at the risk subgroups in 25 CM patients

The results presented above indicated that the m^1^A‐, m^5^C‐ and m^6^A‐related regulators could play an important role in the CM pathogenesis. Therefore, we moved on to investigate the prognostic signature of 46 m^1^A‐, m^5^C‐ and m^6^A‐related regulators in CM. Univariate Cox regression analysis was used to investigate the relationship between the 46 regulators and patient prognosis in TCGA‐SKCM (Figure 4A); we identified a total of 12 regulators significantly related to the OS (Table S3). The regression coefficient of the 12 regulators was computed using the LASSO Cox regression analysis (Figure 4B and Figure 4C). We identified nine regulators: *UNG*, *FMR1*, *MBD4*, *MBD2*, *NEIL1*, *WTAP*, *UHRF2*, *YTHDF1* and *RBM15B* (Figure 4D). To calculate the patient's risk score, a multivariate Cox regression analysis with nine genes was conducted (Table S4). The distribution of the risk score, vital status and expression levels of the corresponding nine regulators in the TCGA data set is shown in Figure 5A and Figure 5B. Using the median risk score to divide patients into the high‐risk and low‐risk groups, the K‐M curve displayed that the risk value could effectively predict survival in CM patients (Figure 5C).

### 3.4 Internal and external validation of the nine‐regulator–related risk model

**FIGURE 6:**
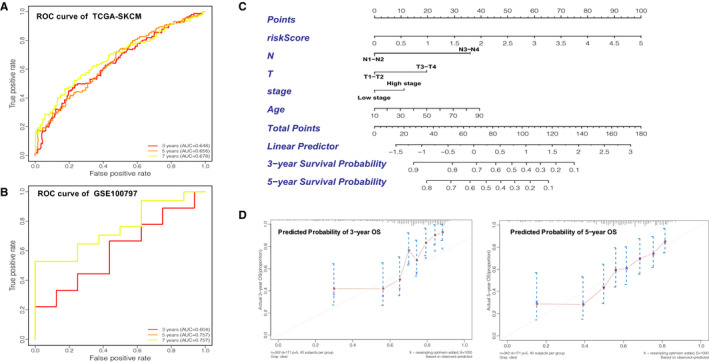
Validation of the prognostic signature of the nine m^1^A‐, m^5^C‐ and m^6^A‐related regulators. (A) AUC of the ROC analysis showed the predicted efficacy of the risk model in the internal training set. (B) The ROC curve for the external validation set. (C) The nomogram of the risk model for predicting the OS probability of CM patients. The whole points projected on the bottom scales indicate the likelihood of 3‐ and 5‐year OS. (D) The calibration plot for the nomogram predicting 3‐year and 5‐year OS. The y‐axis indicates the actual survival, as measured by the K‐M analysis, while the x‐axis shows the nomogram‐predicted survival

In order to examine the performance of the risk model based on nine regulators, we calculated the AUC at 3, 5 and 7 years (Figure 6A). All were greater than 0.64. To further validate the efficacy of the nine‐regulator–related gene signatures, we also performed the above analysis in the GSE100797 data set (external validation set). Based on median risk values, 13 CM patients were classified as high‐risk group and 12 as low‐risk group. As the risk score increased, so did the number of deaths (Figure 5E). The expression pattern of prognostic regulators between the two groups was almost identical to that in the training set (Figure 5G), but there was a shift in the expression pattern of *NEIL1* and *FMR1*, possibly due to the small sample size. The results of the K‐M analysis were consistent with the training set, showing that the patients in the high‐risk group were associated with worse OS (Figure 5F). The ROC curves suggested that the AUCs of the gene signature in the external validation set at 3, 5 and 7 years were 0.604, 0.757 and 0.757, respectively (Figure 6B). The above results indicated that the prognostic signature of m^1^A‐, m^5^C‐ and m^6^A‐related regulators had a reliable validity. Furthermore, the independent prognostic value of the risk score and clinicopathological variables were compared using univariate and multivariate Cox regression analyses (Table 2 and Table S4); the results indicated that risk score, N stage, T stage and age were independent prognostic factors of OS in CM (Figure 5D). To develop a clinically applicable way for the prediction of survival status in CM patients, a nomogram was established to predict the 3‐year and 5‐year OS probability in 468 CM patients (Figure 6C). The calibration plot for nomogram suggested its high predictive accuracy and sensitivity in CM patients (Figure 6D).

**TABLE 2:** Univeriate Cox analysis of prognostic factors for overall survival in TCGA‐SKCM patient

Prognostic variables	Univariate analyses				
	Coef	HR	95% CI		*P*‐value
			low	high	
Risk score	0.58	1.8	1.4	2.3	0.00
Gender	0.01	1.0	0.7	1.4	0.94
Radiotherapy	0.01	1.0	0.7	1.4	0.98
Chemotherapy	0.13	1.1	0.8	1.5	0.41
T	0.66	1.9	1.4	2.7	0.00
N	1.16	3.1	1.9	5.0	0.00
M	0.66	1.9	0.9	4.4	0.11
Stage	−0.54	0.6	0.4	0.8	0.00
Age	0.02	1.0	1.0	1.0	0.00

Note:

Coef the coefficient of table‐regarded features correlated with survival; HR: hazard ratio; 95% CI: 95% confidence interval.

### 3.5 Functional enrichment analyses for the nine‐regulator–related risk subgroups

**FIGURE 7:**
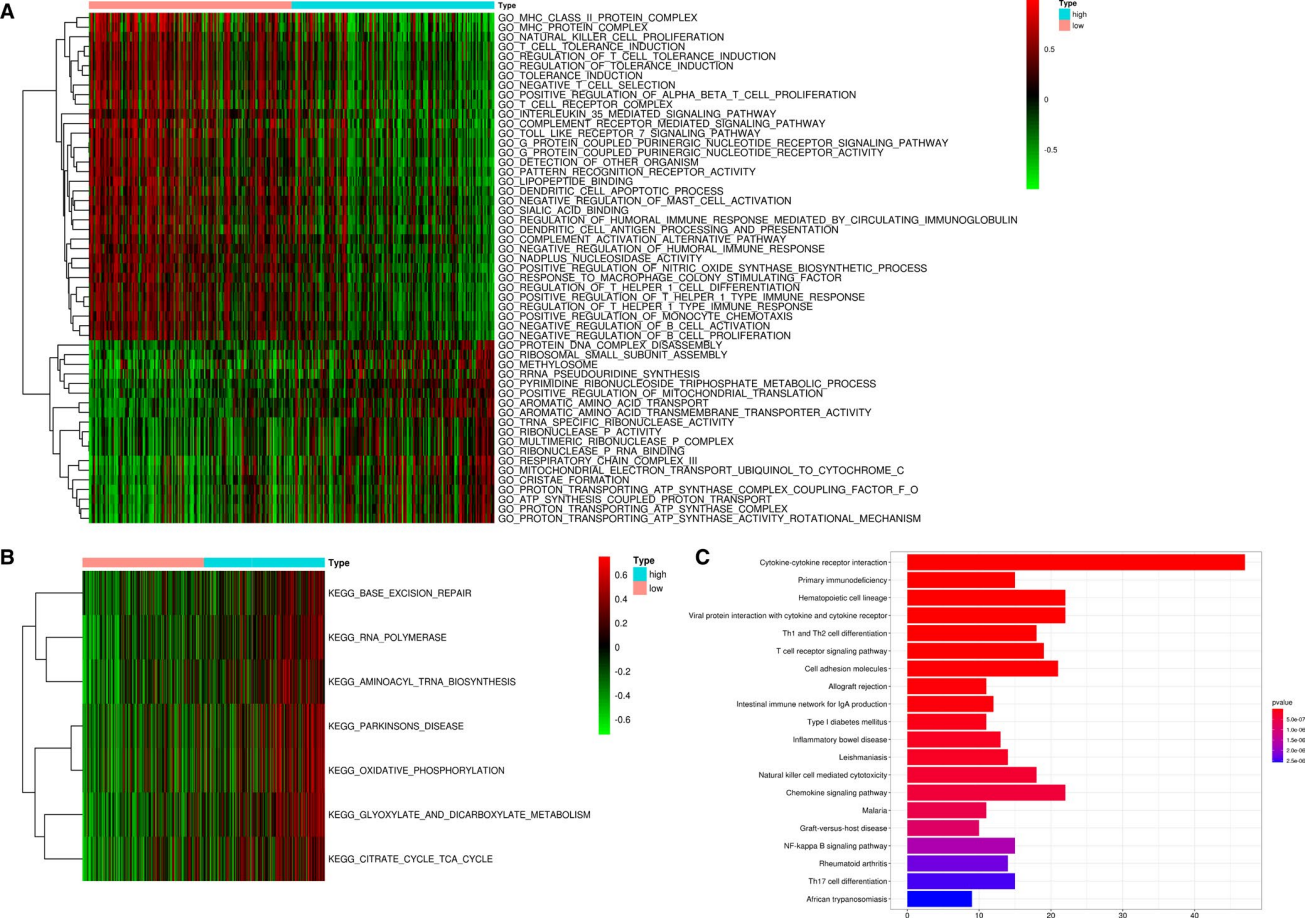
Functional enrichment analyses of the different risk subgroups. (A, B) GO and KEGG term analysis for the different risk subgroups in the TCGA‐SKCM data set. (C) KEGG pathway analysis indicated that DEGs of the two risk subgroups were significantly enriched in cytokine‐cytokine receptor interaction. DEGs: differentially expressed genes

The GSVA enrichment analysis was employed to investigate the underlying biological activities among the high‐ and low‐risk groups. As shown in Figure 7A and Table S6, the high‐risk group was markedly enriched in ‘PROTEIN DNA COMPLEX DISASSEMBLY’, ‘CHROMATIN DISASSEMBLY’ and ‘NuRD COMPLEX’ terms. The GSVA‐KEGG pathways involved in the high‐risk group had a link to immune cell metabolism, as clearly exhibited in Figure 7B such as ‘RNA POLYMERASE’, ‘AMINOACYL tRNA BIOSYNTHESIS’, ‘CITRATE CYCLE TCA CYCLE’ and ‘OXIDATIVE PHOSPHORYLATION’. We analysed the DEGs between the high‐ and low‐risk groups (Figure S3). KEGG pathway analysis was performed to determine the signalling pathways related to DEGs. It identified 39 types of KEGG pathways (*P* adjusted <0.05), especially immune full activation, including cytokine‐cytokine receptor interaction, T‐cell receptor signalling pathway, Th1 and Th2 cell differentiation and cell adhesion molecules (Figure 7C; Figure S4).

### 3.6 Immune infiltration characteristics of TME within nine‐regulator–related risk subgroups

**FIGURE 8:**
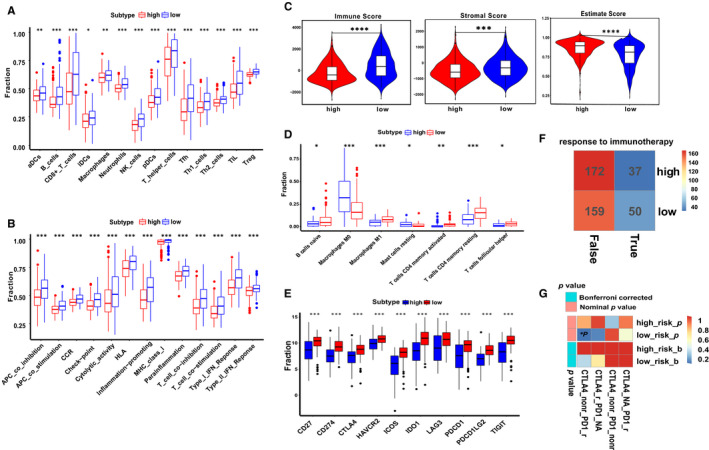
Predicted evaluation of TME characteristics and immunotherapy response. (A, B) Box plot for the TME cells in distinct risk groups derived from CM patients based on the ssGSEA. (C) Immune, stromal and ESTIMATE scores within the low‐ and high‐risk groups. The Wilcoxon signed‐rank test was used to compare the two subgroups. (D) The ICI composition of TME in the two subgroups. (E) The expression levels of well‐known ICPs in the distinct risk subgroups. (F) TIDE estimated the relationship between the response to ICBs treatment and risk subgroups. The low‐risk group showed a better response than the high‐risk group (23.923% > 17.703%). (G) SubMap analysis indicated that the low‐risk group was more likely to respond to anti‐PD‐1 immunotherapy. (*p < 0.05, **p < 0.01, ***p < 0.001 and ****p < 0.0001); ICI: immune cell infiltration; ICPs: immune checkpoints; ICBs: immune checkpoint inhibitors

The ssGSEA was conducted to investigate the ICI pattern related to the risk score based on transcriptome profiling data for 468 SKCM patients from the TCGA database. The low‐risk group was remarkably enriched in innate ICI, which mainly included natural killer cells, macrophages, activated dendritic cells (aDCs), B cells and T cells (Figure 8A). Previously, the low‐risk group identified matched survival advantage in TCGA‐SKCM (Figure 5C). The results of the GSVA showed that the high‐risk group was significantly associated with stromal activation. It has been reported that T‐cell suppression could activate the TME stroma.^23^ Coincidentally, ssGSEA showed a significant decrease in T‐cell activity in the high‐risk group (Figure 8B). Therefore, we speculated that stromal activation in the high‐risk group inhibited the antitumour effect of immune cells. Based on the above analyses, we were surprised to find that the two groups had radically distinct TME cell infiltration characterization. Based on the three scores generated by the ESTIMATE algorithm, we analysed the relationship between scores and high‐/low‐risk groups. As shown in Figure 8C, we could see that high‐ and low‐risk groups had a significant effect on immune, stromal and ESTIMATE scores (all *p* < 0.001). The highest ESTIMATE score was found in the high‐risk group (*p* = 4.1e‐08). The low‐risk group had the highest immune and stromal scores, whereas the high‐risk group had the lowest. We then used a deconvolution algorithm, the CIBERSORT method, for determining the immune cell type in CM, to compare the component differences in immune cells between the high‐ and low‐risk groups. We found that there were significant differences in the compositions of TME cell types between the high‐ and low‐risk groups (Figure 8D). Therefore, we concluded that the immune‐related biological processes and pathways associated with the risk score might result from the observed significant differences in the various immune cell types.

### 3.7 Assessment of immunotherapy response in nine‐regulator–related risk subgroups

A relationship between polymorphisms of human leucocyte antigen (HLA) and the tumour proliferation and immune escape has been studied in the literature.^24^ Pioneering investigations revealed that immunotherapy targeting immune checkpoints (ICPs) offered great hope for the clinical treatment of human cancers. Given that the HLA was differentially expressed between high‐ and low‐risk groups (Figure 8E), it was necessary to analyse the correlation between the expression levels of several well‐known immune checkpoint molecules (ICMs) in distinct risk subgroups. In the high‐risk group, ICMs were lowly expressed, suggesting that the low‐risk group could be more suitable for immunotherapy. CTLA‐4 and PD‐1 as well‐known ICBs improved OS in especially CM patients with metastatic or advanced disease.^25^, ^26^ Potential response to immunotherapy in patients from the different risk subgroups was modelled on TIDE instructions, and T‐cell dysfunction and rejection were used to predict the performance of ICBs in the two subgroups. We found that the low‐risk group (23.923%, 50/209) was significantly more eligible for immunotherapy compared with the high‐risk group (17.703%, 37/209) (Figure 8F). SubMap is an unsupervised clustering method that can match subclasses in two independent gene expression data sets.^27^ Herein, an inspection of the SubMap analysis data in Figure 8G revealed that the low‐risk group was more likely to respond to anti‐PD‐1 therapy (*p* < 0.05).

## 4 DISCUSSION

CM is a potentially deadly form of skin cancer, and its pathogenesis remains controversial.^25^ As a consequence of the molecular heterogeneity, single prognostic factors sometimes fail in risk stratification and clinical outcome estimations. The development of effective genetic signatures that integrate multiple prognostic indicators to facilitate the prediction of survival in CM patients is urgently needed. This study constructed and validated a novel prognostic model for CM based on m^1^A‐, m^5^C‐ and m^6^A‐related regulators by using the TCGA database.

We summarized the mutation frequency and CNV alteration in TCGA‐SKCM samples. Furthermore, SNV and CNV of regulators could affect the expression of crucial regulatory molecules in CM patients. Mounting evidence suggests that RNA modification–related regulators, involving m^1^A, m^5^C and m^6^A, could serve as biomarkers in several malignancies.^28^ Specifically, Chen et al. demonstrated that *WTAP* as m^6^A‐related regulator could mediate cell cycle regulation. Silencing the expression of *WTAP* could affect the expression of ETS1 in HCC. ETS1 was recognized as the downstream molecular target.^29^ Several studies have reported that *MBD4*, *RBM15B*, *YTHDF1* and *NEIL1* were associated with OS and clinical features in the various tumours, including uveal melanoma (UM),^30^ melanoma^31^ and head and neck squamous cell carcinoma.^32^ *MBD4* could act as a purposeful biomarker and a latent target in UM patients. *RBM15B* was shown to bind CDK11‐cyclin L to inhibit the cell cycle and suppress the UM invasion and metastasis.^30^ *YTHDF1* was shown to interact with genes related to p53 signalling, such as CDK2, CDK1, RRM2, CCNB1 and CHEK1, resulting in the development of melanoma.^33^ Because CNV alterations could affect gene expression levels via dose compensation effects, we analysed the correlation between those related regulatory gene mRNA expression levels and CNV patterns (Figure S1). Distinct associations were found between mRNA expression levels and CNV patterns in 468 CM samples. For the 46 regulatory genes, increased copy numbers for 45 genes were correlated with higher mRNA expression, while deletions led to decreasing mRNA expression. Collectively, m^1^A‐, m^5^C‐ and m^6^A‐related regulatory gene expression levels substantially correlated with the clinical TNM stage and prognosis in CM.

A novel prognostic signature of m^1^A‐, m^5^C‐ and m^6^A‐related regulators identified could precisely distinguish the OS of CM patients. The classification ability of risk model was verified respectively on TCGA‐SKCM training set and GSE00797 data set. It is now well established from previous studies that the expression level, genetic mutation and molecular subtype of m^6^A‐related regulators had non‐negligible impacts on the development and progression of CM.^22^, ^24^, ^28^ A risk model on m^6^A‐related regulators suggested that degradation‐enhancing molecular subtype was related to favourable prognosis in CM.^34^ Another research demonstrated that m^6^A‐related regulators could regulate m^6^A‐related lncRNAs to affect CM prognosis.^35^ Therefore, comprehensive analysis of m^1^A‐, m^5^C‐ and m^6^A‐related regulatory gene signatures is important to understand the complex heterogeneity in CM.

TME plays a vital role in the therapeutic resistance in CM patients.^14^ In particular, the specific mechanism of interaction between TME and immune cell infiltration (ICI) significantly influences CM prognosis.^36^, ^37^ In this study, the high‐risk group with survival disadvantage was rich in immune full activation pathway, which might be resulted from T‐cell suppression. Previous studies revealed that the different m^6^A modification patterns could activate stromal and mediate therapeutic resistance to ICBs.^22^, ^38^ On the basis of these findings, Hu et al. comprehensively summarized the ICI in TCGA‐SKCM and verified the correlation between high ICI and better prognosis.^39^ Subsequently, the results of CIBERSORT and ESTIMATE algorithms in accordance with the reviewing literatures revealed low‐risk group had higher ICI and stromal score. Likewise, the infiltration level of B cells, CD8^+^ T cells, TIL and HLA was significantly higher in the low‐risk group; however, some specific immune cell types and ICBs, including CD4^+^ memory T cells, CD4^+^ T activated cells, macrophage (M1) and CTLA‐4, were lower. The regulator‐related low‐risk group was more suitable for immunotherapy and displayed relatively better immunogenicity. It is somewhat surprising that low‐risk group was sensitive to anti‐PD‐1 but anti‐CTLA‐4 immunotherapy. In a previous study, a four‐gene tumour immune‐relevant (TIR) signature was identified. The TIR signature could predict the response to ipilimumab and the survival. Notably, the predictive power of the TIR signature was higher than that of other biomarkers. The expression levels of four genes were positively associated with the infiltration levels of CD8+ T cells and CD4+ T cells. They also found significant correlations of these four genes with the mRNA levels of CTLA‐4 and PD‐L1. Therefore, the increased T‐lymphocyte infiltration is likely a major cause of resistance to anti‐CTLA‐4 immunotherapy.^40^ These results corroborate the findings of the previous work in the immune‐related^41^ and tumour mutation burden (TMB)–related gene signature of CM.^42^ Indeed, the m^1^A‐, m^5^C‐ and m^6^A‐related prognostic signature as an attractive determinant of immunogenicity may contribute to deeply explore the potential mechanism of immune‐resistant CM.

Collectively, our study summarized the signature of m^1^A‐, m^5^C‐ and m^6^A‐related regulators in CM and evaluated the associations with OS. Furthermore, the most clinically relevant finding is the establishment of regulator‐related risk prediction model, which could be an alternative classifier for more accurate and efficient immunotherapy in patients with CM. Additionally, the potential relevance of nine m^1^A‐, m^5^C‐ and m^6^A‐related regulatory gene signature to the ICI provides support for novel ICP discovery. Despite these promising results, limitations remain. Investigation of the specific molecular subtypes of m^1^A‐, m^5^C‐ and m^6^A‐related regulators is required. The samples in our study are only derived from the two data sets. The predictive ability of the risk model may be limited because of the limited corresponding sample data. More samples are required to validate the generalization of this risk model. Further studies should take these factors into account. Besides, further work is required to shed light on the specific immune regulation mechanism of nine prognostic genes in CM.

## CONFLICT OF INTEREST

The authors confirm that there are no conflicts of interest.

## AUTHOR CONTRIBUTION

**xian rui Wu:** Conceptualization (lead); Data curation (lead); Formal analysis (lead); Funding acquisition (equal); Investigation (lead); Methodology (lead); Project administration (equal); Resources (equal); Software (lead); Supervision (lead); Validation (lead); Visualization (lead); Writing‐original draft (lead); Writing‐review & editing (lead). **zheng Chen:** Data curation (equal); Formal analysis (equal); Methodology (equal); Validation (equal); Visualization (equal); Writing‐original draft (equal). **yang Liu:** Data curation (equal); Formal analysis (equal); Methodology (equal); Software (equal); Visualization (equal); Writing‐original draft (equal). **zi zi Chen:** Formal analysis (equal); Investigation (equal); Methodology (equal); Validation (equal); Writing‐original draft (equal). **zhi zhao Chen:** Formal analysis (equal); Investigation (equal); Methodology (equal); Software (equal); Validation (equal). **feng jie Tang:** Formal analysis (equal); Investigation (equal); Software (equal); Visualization (equal). **jing jing Li:** Data curation (equal); Formal analysis (equal); Methodology (equal); Validation (equal). **jun lin Liao:** Data curation (equal); Formal analysis (equal); Methodology (equal); Visualization (equal). **ke Cao:** Data curation (equal); Methodology (equal); Project administration (equal); Supervision (equal); Validation (equal); Writing‐review & editing (equal). **Xiang Chen:** Conceptualization (equal); Data curation (equal); Methodology (equal); Project administration (equal); Resources (equal); Supervision (equal). **Jianda Zhou:** Conceptualization (equal); Data curation (equal); Funding acquisition (equal); Project administration (lead); Resources (equal); Supervision (lead); Writing‐original draft (equal); Writing‐review & editing (equal).

## Supporting information

Figure S1

Figure S2

Figure S3

Figure S4

Figure S5

Table S1‐S6

Supplementary Material

## Data Availability

The data analysed in this study were derived from the public domain resources: http://gdc.cancer.gov and http://www.ncbi.nlm.nih.gov/geo/.
