# Supplementary figures and images for: Prognostic signature and immune efficacy of m1A‐, m5C‐ and m6A‐related regulators in cutaneous melanoma
Source: J Cell Mol Med. 2021 Jul 21;25(17):8405–18. doi: 10.1111/jcmm.16800 (PMC8419166; doi:10.1111/jcmm.16800)

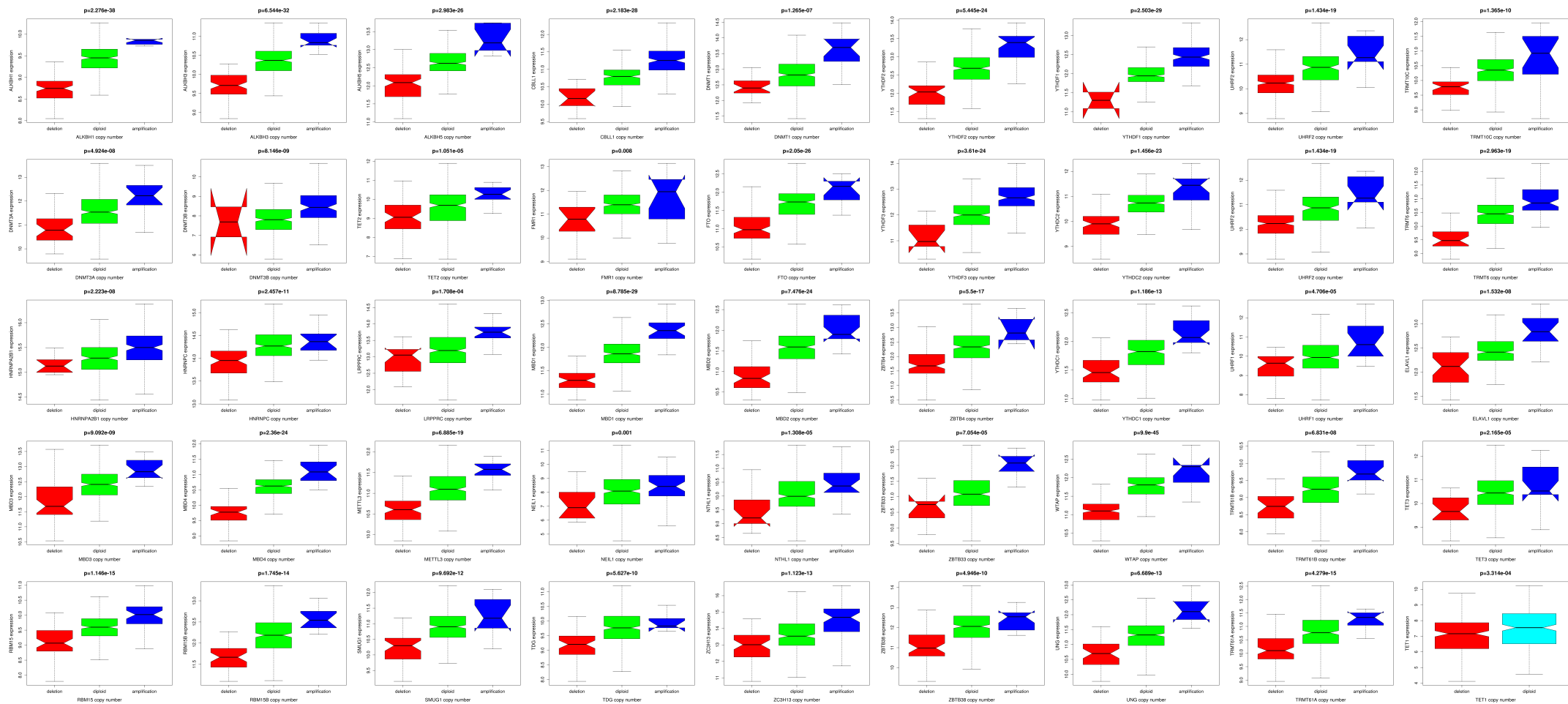

**Figure S1 Expression of 45 regulators in different CNV patterns.**

Supplement: Supplementary file 1 — Figure S1 [file JCMM-25-8405-s007.pdf]

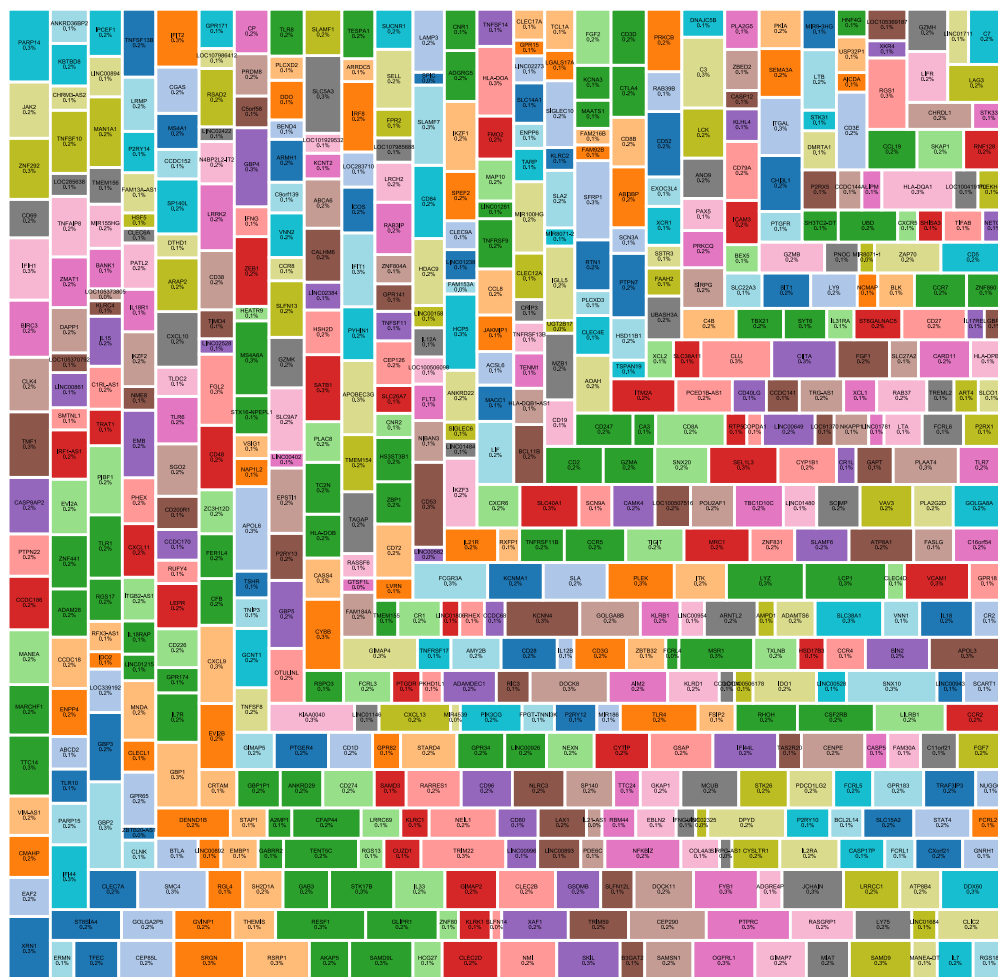

**Figure S3 Gene expression treemap of DEGs from the regulators-related risk subgroups.**

Supplement: Supplementary file 3 — Figure S3 [file JCMM-25-8405-s006.pdf]

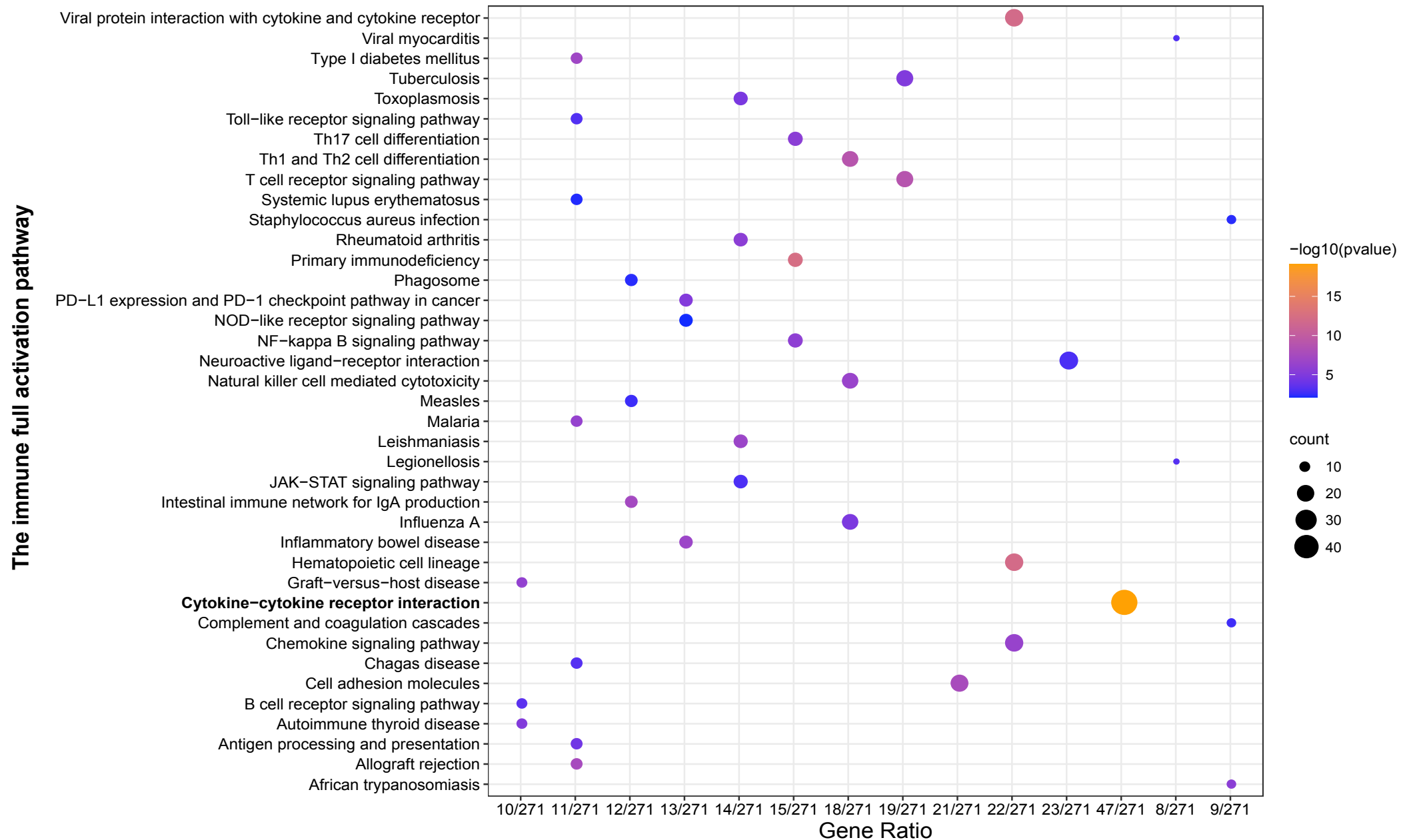

**Figure S4 Scatter plot of DEGs enriched 37 KEGG pathways.**

Supplement: Supplementary file 4 — Figure S4 [file JCMM-25-8405-s003.pdf]

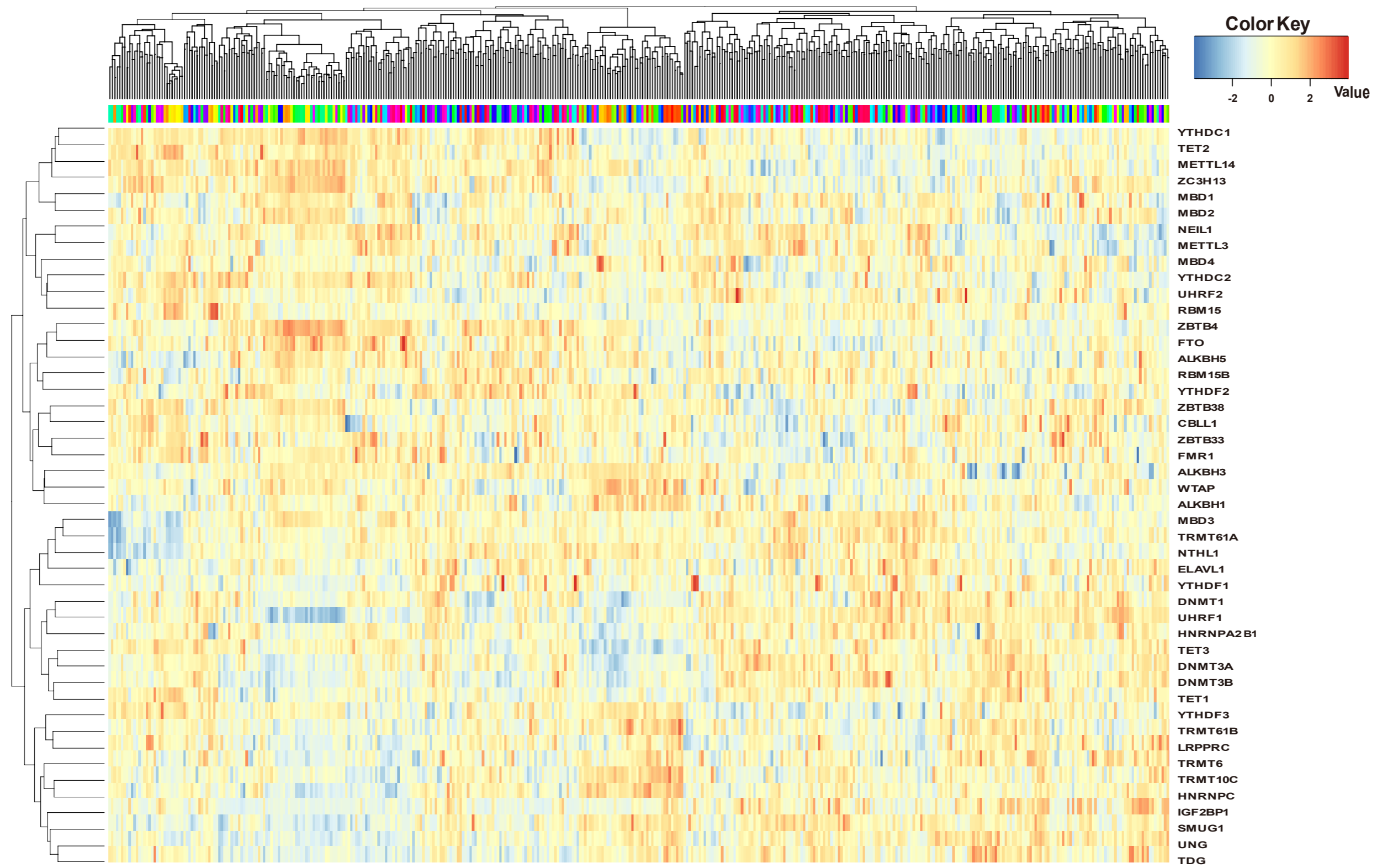

**Figure S5 Heatmap of 46 regulatory genes in TCGA-SKCM datasets.**

Supplement: Supplementary file 5 — Figure S5 [file JCMM-25-8405-s004.pdf]
