# Supplementary material for: Prognostic signature and immune efficacy of m1A‐, m5C‐ and m6A‐related regulators in cutaneous melanoma
Source: J Cell Mol Med. 2021 Jul 21;25(17):8405–18. doi: 10.1111/jcmm.16800 (PMC8419166; doi:10.1111/jcmm.16800)
Supplement: Supplementary file 2 — Figure S2 [file JCMM-25-8405-s002.pdf]

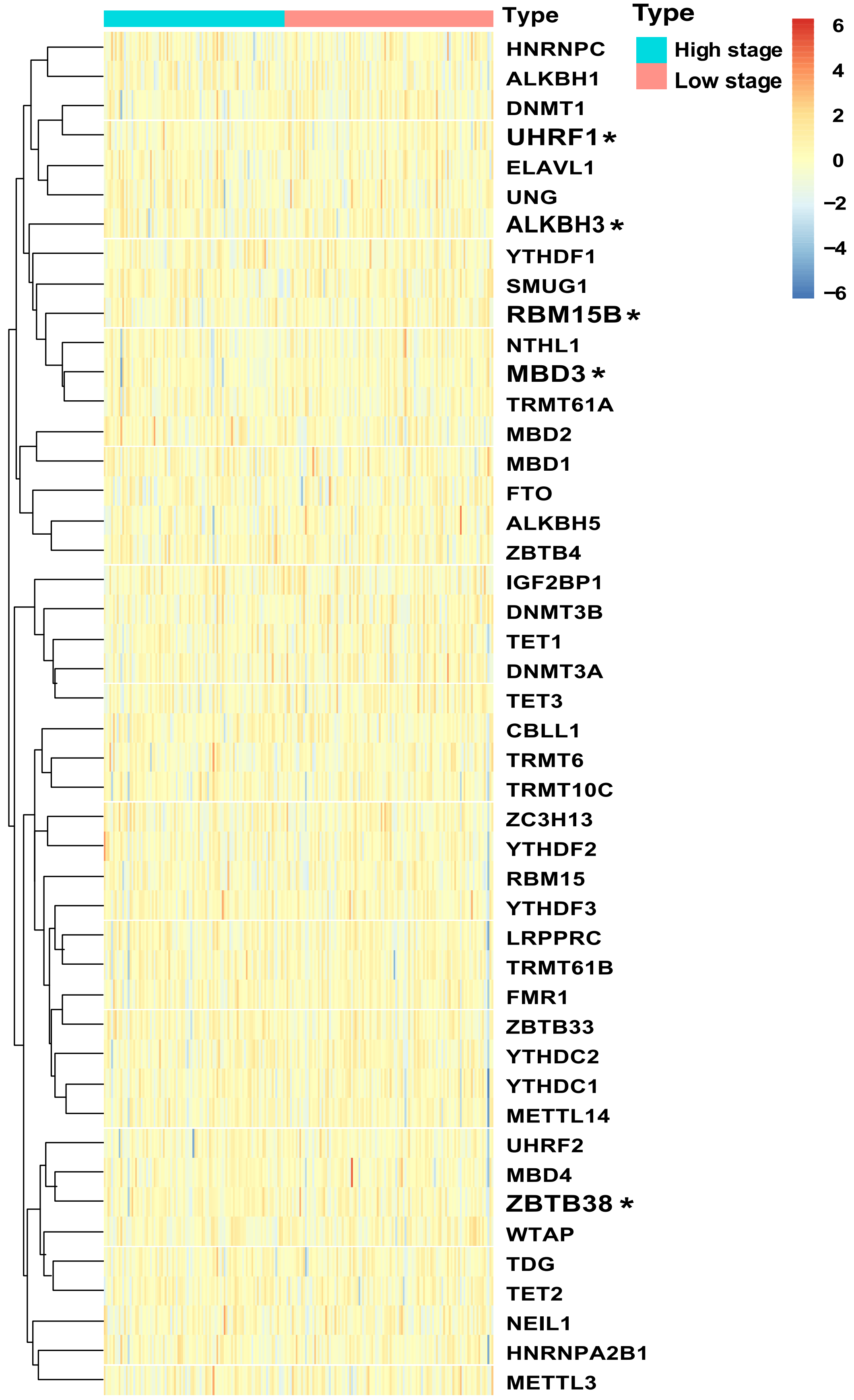


**Figure S2 Heatmap for the expression levels of 46 regulators within different clinical TNM stages.**
