# Supplementary material for: Prognostic signature and immune efficacy of m1A‐, m5C‐ and m6A‐related regulators in cutaneous melanoma
Source: J Cell Mol Med. 2021 Jul 21;25(17):8405–18. doi: 10.1111/jcmm.16800 (PMC8419166; doi:10.1111/jcmm.16800)
Supplement: Supplementary file 6 — Table S1‐S6 [file JCMM-25-8405-s001.docx]

**Supplementary Tables：**

| **Symbol** | **Description** |
| --- | --- |
| ALKBH1 | alkB homolog 1, histone H2A dioxygenase |
| ALKBH3 | alkB homolog 3, alpha-ketoglutaratedependent dioxygenase |
| ALKBH5 | alkB homolog 5, RNA demethylase |
| CBLL1 | Cbl proto-oncogene like 1 |
| DNMT1 | DNA methyltransferase 1 |
| DNMT3A | DNA methyltransferase 3 alpha |
| DNMT3B | DNA methyltransferase 3 beta |
| ELAVL1 | ELAV like RNA binding protein 1 |
| FMR1 | fragile X mental retardation 1 |
| FTO | FTO, alpha-ketoglutarate dependent dioxygenase |
| HNRNPA2B | heterogeneous nuclear ribonucleoprotein A2/B1 |
| HNRNPC | heterogeneous nuclear ribonucleoprotein C (C1/C2) |
| IGF2BP1 | insulin like growth factor 2 mRNA binding protein 1 |
| LRPPRC | leucine rich pentatricopeptide repeat containing |
| MBD1 | methyl-CpG binding domain protein 1 |
| MBD2 | methyl-CpG binding domain protein 2 |
| MBD3 | methyl-CpG binding domain protein 3 |
| MBD4 | methyl-CpG binding domain 4, DNA glycosylase |
| METTL14 | methyltransferase like 14 |
| METTL3 | methyltransferase like 3 |
| NEIL1 | nei like DNA glycosylase 1 |
| NTHL1 | nth like DNA glycosylase 1 |
| RBM15 | RNA binding motif protein 15 |
| RBM15B | RNA binding motif protein 15B |
| SMUG1 | single-strand-selective monofunctional uracil-DNA glycosylase 1 |
| TDG | thymine DNA glycosylase |
| TET1 | tet methylcytosine dioxygenase 1 |
| TET2 | tet methylcytosine dioxygenase 2 |
| TET3 | tet methylcytosine dioxygenase 3 |
| TRMT10C | tRNA methyltransferase 10C, mitochondrial RNase P subunit |
| TRMT6 | tRNA methyltransferase 6 |
| TRMT61A | tRNA methyltransferase 61A |
| TRMT61B | tRNA methyltransferase 61B |
| UHRF1 | ubiquitin like with PHD and ring finger domains 1 |
| UHRF2 | ubiquitin like with PHD and ring finger domains 2 |
| UNG | uracil DNA glycosylase |
| WTAP | Wilms tumor 1 associated protein |
| YTHDC1 | YTH domain containing 1 |
| YTHDC2 | YTH domain containing 2 |
| YTHDF1 | YTH N6-methyladenosine RNA binding protein 1 |
| YTHDF2 | YTH N6-methyladenosine RNA binding protein 2 |
| YTHDF3 | YTH N6-methyladenosine RNA binding protein 3 |
| ZBTB33 | zinc finger and BTB domain containing 33 |
| ZBTB38 | zinc finger and BTB domain containing 38 |
| ZBTB4 | zinc finger and BTB domain containing 4 |
| ZC3H13 | zinc finger CCCH-type containing 13 |

**Table S1 Basic information of 46 candidate regulators in the study.**

| **SNV-mediated** | |  | **CNV-mediated** | | | |
| --- | --- | --- | --- | --- | --- | --- |
| **regulators** | ***P* value** |  | | **regulators** | | ***P* value** |
| ***DNMT3B** | **0.032** |  | WTAP | | 9.90E-45 | |
| ***DNMT1** | **0.033** |  | ALKBH1 | | 2.28E-38 | |
| ***HNRNPC** | **0.045** |  | ALKBH3 | | 6.54E-32 | |
| ***UNG** | **0.049** |  | YTHDF1 | | 2.50E-29 | |
| ZBTB4 | 0.074 |  | MBD1 | | 8.79E-29 | |
| IGF2BP1 | 0.123 |  | CBLL1 | | 2.18E-28 | |
| ZC3H13 | 0.134 |  | FTO | | 2.05E-26 | |
| NEIL1 | 0.153 |  | ALKBH5 | | 2.98E-26 | |
| FMR1 | 0.174 |  | MBD4 | | 2.36E-24 | |
| MBD4 | 0.183 |  | YTHDF3 | | 3.61E-24 | |
| ZBTB33 | 0.234 |  | YTHDF2 | | 5.44E-24 | |
| YTHDF3 | 0.269 |  | MBD2 | | 7.48E-24 | |
| METTL14 | 0.304 |  | YTHDC2 | | 1.46E-23 | |
| YTHDF1 | 0.304 |  | METTL14 | | 4.44E-23 | |
| ALKBH3 | 0.309 |  | UHRF2 | | 1.43E-19 | |
| NTHL1 | 0.318 |  | TRMT6 | | 2.96E-19 | |
| UHRF1 | 0.320 |  | METTL3 | | 6.89E-19 | |
| WTAP | 0.323 |  | ZBTB4 | | 5.50E-17 | |
| TDG | 0.327 |  | RBM15 | | 1.15E-15 | |
| MBD1 | 0.381 |  | TRMT61A | | 4.28E-15 | |
| METTL3 | 0.385 |  | RBM15B | | 1.74E-14 | |
| YTHDC1 | 0.414 |  | ZC3H13 | | 1.12E-13 | |
| TRMT10C | 0.45 |  | YTHDC1 | | 1.19E-13 | |
| DNMT3A | 0.456 |  | UNG | | 6.69E-13 | |
| TRMT61A | 0.491 |  | SMUG1 | | 9.69E-12 | |
| ZBTB38 | 0.517 |  | HNRNPC | | 2.46E-11 | |
| CBLL1 | 0.568 |  | TRMT10C | | 1.37E-10 | |
| ELAVL1 | 0.574 |  | ZBTB38 | | 4.95E-10 | |
| YTHDF2 | 0.615 |  | TDG | | 5.63E-10 | |
| TRMT6 | 0.619 |  | DNMT3B | | 8.15E-09 | |
| FTO | 0.641 |  | MBD3 | | 9.09E-09 | |
| HNRNPA2B1 | 0.680 |  | ELAVL1 | | 1.53E-08 | |
| MBD2 | 0.702 |  | HNRNPA2B1 | | 2.22E-08 | |
| TET1 | 0.710 |  | DNMT3A | | 4.92E-08 | |
| TRMT61B | 0.712 |  | TRMT61B | | 6.83E-08 | |
| TET2 | 0.723 |  | DNMT1 | | 1.27E-07 | |
| LRPPRC | 0.727 |  | TET2 | | 1.05E-05 | |
| TET3 | 0.822 |  | NTHL1 | | 1.31E-05 | |
| RBM15B | 0.857 |  | TET3 | | 2.17E-05 | |
| ALKBH1 | 0.867 |  | UHRF1 | | 4.71E-05 | |
| SMUG1 | 0.874 |  | ZBTB33 | | 7.05E-05 | |
| YTHDC2 | 0.890 |  | LRPPRC | | 0.00017 | |
| RBM15 | 0.956 |  | TET1 | | 0.00033 | |
| MBD3 | 1 |  | NEIL1 | | 0.00143 | |
| - | - |  | FMR1 | | 0.00758 | |
| - | - |  | **^#^IGF2BP1** | | **0.08056** | |

**Table S2** **Expression of SNV- and CNV- mediated regulators in TCGA-SKCM.**

Note: Bold values indicate **P* < 0.05 with significantly statistical difference, while ^#^*P* > 0.05 represents no statistical difference.

| **regulators** | **univariate Cox regression analysis** | | | | | |
| --- | --- | --- | --- | --- | --- | --- |
|  | **coefficient** | **HR** | **95% CI of HR** | | | ***P* value** |
|  |  |  | **low** | **high** | |  |
| *****MBD4** | -0.56 | 0.57 | 0.45 | | 0.74 | **0.00** |
| ****UNG** | 0.37 | 1.50 | 1.20 | | 1.80 | **0.00** |
| ****RBM15B** | 0.46 | 1.60 | 1.20 | | 2.10 | **0.00** |
| ****MBD2** | -0.4 | 0.67 | 0.51 | | 0.88 | **0.00** |
| ****ZBTB38** | -0.21 | 0.81 | 0.70 | | 0.94 | **0.00** |
| ****FMR1** | -0.29 | 0.75 | 0.61 | | 0.91 | **0.00** |
| ****WTAP** | -0.39 | 0.68 | 0.51 | | 0.90 | **0.01** |
| ****ELAVL1** | 0.56 | 1.70 | 1.20 | | 2.60 | **0.01** |
| ****YTHDF1** | 0.38 | 1.50 | 1.10 | | 1.90 | **0.01** |
| ***UHRF2** | -0.21 | 0.81 | 0.68 | | 0.97 | **0.02** |
| ***METTL14** | -0.3 | 0.74 | 0.57 | | 0.97 | **0.03** |
| ***NEIL1** | -0.12 | 0.89 | 0.80 | | 0.99 | **0.03** |
| METTL3 | -0.26 | 0.77 | 0.59 | | 1.00 | 0.06 |
| DNMT3B | 0.13 | 1.10 | 0.99 | | 1.30 | 0.07 |
| ZC3H13 | 0.12 | 1.10 | 0.99 | | 1.30 | 0.08 |
| ALKBH5 | 0.24 | 1.30 | 0.97 | | 1.70 | 0.08 |
| UHRF1 | 0.12 | 1.10 | 0.98 | | 1.30 | 0.09 |
| SMUG1 | 0.2 | 1.20 | 0.97 | | 1.50 | 0.09 |
| YTHDF2 | 0.19 | 1.20 | 0.93 | | 1.60 | 0.15 |
| IGF2BP1 | 0.029 | 1.00 | 0.99 | | 1.10 | 0.16 |
| TET2 | -0.092 | 0.91 | 0.80 | | 1.00 | 0.18 |
| MBD1 | 0.21 | 1.20 | 0.87 | | 1.70 | 0.24 |
| YTHDC1 | -0.23 | 0.80 | 0.54 | | 1.20 | 0.26 |
| TRMT61A | 0.11 | 1.10 | 0.91 | | 1.40 | 0.29 |
| MBD3 | 0.10 | 1.10 | 0.90 | | 1.40 | 0.33 |
| HNRNPC | 0.19 | 1.20 | 0.81 | | 1.80 | 0.35 |
| DNMT3A | 0.092 | 1.10 | 0.91 | | 1.30 | 0.35 |
| ALKBH3 | 0.14 | 1.10 | 0.86 | | 1.50 | 0.35 |
| ZBTB33 | -0.11 | 0.90 | 0.71 | | 1.10 | 0.35 |
| ZBTB4 | -0.099 | 0.91 | 0.73 | | 1.10 | 0.38 |
| RBM15 | -0.14 | 0.87 | 0.63 | | 1.20 | 0.39 |
| FTO | 0.11 | 1.10 | 0.87 | | 1.40 | 0.40 |
| TET3 | 0.075 | 1.10 | 0.90 | | 1.30 | 0.42 |
| LRPPRC | 0.091 | 1.10 | 0.87 | | 1.40 | 0.45 |
| TRMT61B | -0.068 | 0.93 | 0.73 | | 1.20 | 0.59 |
| HNRNPA2B1 | -0.08 | 0.92 | 0.61 | | 1.40 | 0.70 |
| DNMT1 | 0.046 | 1.00 | 0.83 | | 1.30 | 0.70 |
| TRMT10C | -0.029 | 0.97 | 0.76 | | 1.20 | 0.82 |
| TDG | -0.022 | 0.98 | 0.77 | | 1.20 | 0.86 |
| CBLL1 | 0.026 | 1.00 | 0.76 | | 1.40 | 0.87 |
| TRMT6 | 0.01 | 1.00 | 0.82 | | 1.20 | 0.92 |
| TET1 | -0.0063 | 0.99 | 0.88 | | 1.10 | 0.92 |
| YTHDF3 | 0.0032 | 1.00 | 0.81 | | 1.20 | 0.98 |
| ALKBH1 | -0.00015 | 1.00 | 0.72 | | 1.40 | 1.00 |

**Table S3** **Univariate Cox regression analysis of the 46 regulators in TCGA-SKCM.**

Note: Bold values indicate **P* < 0.05, ***P* < 0.01, and ****P* < 0.001 with significantly statistical difference; HR: Hazard Ratio; CI: Confident Interval.

| **regulators** | **multivariate Cox regression analysis** | | | | | |
| --- | --- | --- | --- | --- | --- | --- |
|  | **coefficient** | **HR** | **95% CI of HR** | | | ***P* value** |
|  |  |  | **low** | **high** | |  |
| UNG | 0.21 | 1.20 | 0.95 | | 1.60 | 0.11 |
| FMR1 | -0.12 | 0.89 | 0.69 | | 1.10 | 0.34 |
| MBD4 | -0.36 | 0.70 | 0.52 | | 0.94 | 0.02 |
| MBD2 | -0.14 | 0.87 | 0.64 | | 1.20 | 0.38 |
| NEIL1 | -0.07 | 0.93 | 0.83 | | 1.10 | 0.25 |
| WTAP | -0.04 | 0.96 | 0.69 | | 1.30 | 0.83 |
| UHRF2 | -0.05 | 0.95 | 0.79 | | 1.20 | 0.63 |
| YTHDF1 | 0.10 | 1.10 | 0.78 | | 1.60 | 0.57 |
| RBM15B | 0.07 | 1.10 | 0.76 | | 1.50 | 0.70 |

**Table S4 Multivariate Cox regression analysis for prognostic regulators.**

Note: HR: Hazard Ratio; CI: Confident Interval.

| **Clinical variables** | **Total**  **(N=25)** | **Risk-group** | | ***P* value** |
| --- | --- | --- | --- | --- |
|  |  | **high(n=12)** | **low(n=13)** |  |
| **Sample timepoint^a^** |  |  |  | 0.513 |
| After Ipilimumab/Before TILs | 1 (4.0%) | 0 (0%) | 1 (7.7%) |  |
| Before TILs | 20 (80.0%) | 10 (83.3%) | 10 (76.9%) |  |
| Before Vemurafenib/Before TILs | 1 (4.0%) | 1 (8.3%) | 0 (0%) |  |
| During Vemurafenib/Before TILs | 3 (12.0%) | 1 (8.3%) | 2 (15.4%) |  |
| **AJCC stage** |  |  |  | 0.511 |
| M1a | 1 (4.0%) | 0 (0%) | 1 (7.7%) |  |
| M1b | 3 (12.0%) | 1 (8.3%) | 2 (15.4%) |  |
| M1c | 21 (84.0%) | 11 (91.7%) | 10 (76.9%) |  |
| **Type of lesion^b^** |  |  |  | 0.255 |
| IA | 2 (8.0%) | 2 (16.7%) | 0 (0%) |  |
| IM | 1 (4.0%) | 1 (8.3%) | 0 (0%) |  |
| LN | 12 (48.0%) | 4 (33.3%) | 8 (61.5%) |  |
| pleura | 1 (4.0%) | 1 (8.3%) | 0 (0%) |  |
| SC | 8 (32.0%) | 4 (33.3%) | 4 (30.8%) |  |
| Missing | 1 (4.0%) | 0 (0%) | 1 (7.7%) |  |
| **Type of primary** |  |  |  | 0.261 |
| mucosa | 2 (8.0%) | 2 (16.7%) | 0 (0%) |  |
| skin | 17 (68.0%) | 8 (66.7%) | 9 (69.2%) |  |
| unknown | 6 (24.0%) | 2 (16.7%) | 4 (30.8%) |  |
| **RECIST^c^** |  |  |  | 0.288 |
| CR | 5 (20.0%) | 1 (8.3%) | 4 (30.8%) |  |
| PD | 5 (20.0%) | 2 (16.7%) | 3 (23.1%) |  |
| PR | 5 (20.0%) | 2 (16.7%) | 3 (23.1%) |  |
| SD | 10 (40.0%) | 7 (58.3%) | 3 (23.1%) |  |

**Table S5 Different clinicopathological features between the high-risk group and low-risk group in GSE100797 dataset.**

Note：^a^TILs tumor infiltrating lymphocytes; ^b^IA intra-abdominal; IM intra-muscular; LN lymph node; SC subcutaneous; ^c^CR complete responders; PR partial responders; SD stable disease; PD progressive disease；

| **GO_terms** | **logFC** | **AveExpr** | **t** | ***P*-Value** | **adj *P* Val** |
| --- | --- | --- | --- | --- | --- |
| GO_PROTEIN_DNA_COMPLEX_DISASSEMBLY | -0.21761 | -0.04964 | -10.4578 | 6.32E-23 | 4.32E-19 |
| GO_CHROMATIN_DISASSEMBLY | -0.20554 | -0.04135 | -10.3206 | 1.99E-22 | 1.36E-18 |
| GO_NURD_COMPLEX | -0.20922 | -0.06052 | -10.0838 | 1.41E-21 | 9.61E-18 |
| GO_HISTONE_ARGININE_METHYLATION | -0.20949 | -0.02752 | -9.47645 | 1.88E-19 | 1.28E-15 |
| GO_CRISTAE_FORMATION | -0.20862 | -0.04767 | -8.79827 | 3.54E-17 | 2.39E-13 |
| GO_RRNA_PSEUDOURIDINE_SYNTHESIS | -0.24057 | -0.04768 | -8.79213 | 3.71E-17 | 2.50E-13 |
| GO_POSITIVE_REGULATION_OF_TRANSCRIPTION_OF_NUCLEOLAR_LARGE_RRNA_BY_RNA_POLYMERASE_I | -0.21248 | -0.04759 | -8.7686 | 4.43E-17 | 2.99E-13 |
| GO_RIBOSOMAL_SMALL_SUBUNIT_ASSEMBLY | -0.22251 | -0.05055 | -8.612 | 1.43E-16 | 9.59E-13 |
| GO_ATP_SYNTHESIS_COUPLED_PROTON_TRANSPORT | -0.20796 | -0.05297 | -7.98838 | 1.30E-14 | 8.65E-11 |
| GO_RESPIRATORY_CHAIN_COMPLEX_III | -0.23799 | -0.05298 | -7.97723 | 1.41E-14 | 9.35E-11 |
| GO_MITOCHONDRIAL_ELECTRON_TRANSPORT_UBIQUINOL_TO_CYTOCHROME_C | -0.21749 | -0.04692 | -7.62273 | 1.64E-13 | 1.08E-09 |
| GO_PROTON_TRANSPORTING_ATP_SYNTHASE_ACTIVITY_ROTATIONAL_MECHANISM | -0.22231 | -0.05257 | -7.45578 | 5.08E-13 | 3.32E-09 |
| GO_METHYLOSOME | -0.21124 | -0.04629 | -7.04361 | 7.61E-12 | 4.88E-08 |

**Table S6 The GSVA enrichment analysis of** **the regulators-related risk model.**

Note: AveExp: average expression; t: t-test; adj *P*-Val: adjust *P*-value;
