# Supplementary material for: Prognostic signature and immune efficacy of m1A‐, m5C‐ and m6A‐related regulators in cutaneous melanoma
Source: J Cell Mol Med. 2021 Jul 21;25(17):8405–18. doi: 10.1111/jcmm.16800 (PMC8419166; doi:10.1111/jcmm.16800)
Supplement: Supplementary file 7 — Supplementary Material [file JCMM-25-8405-s005.docx]

**Supplementary Figure Legends：**

**Figure S1 Expression of 45 regulators in different CNV patterns.** Increased copy numbers of 45 regulators showed high expression, while deletions presented low. No amplification was detected in *TET1* (on the bottom right corner). Results showed that various CNV patterns were significantly associated with 45-regulators (*ALKBH1, ALKBH3, ALKBH5, CBLL1, DNMT1, DNMT3A, DNMT3B, ELAVL1, FMR1, FTO, HNRNPA2B1, HNRNPC, LRPPRC, MBD1, MBD2, MBD3, MBD4, METTL3, METTL14, NEIL1, NTHL1, RBM15B, RBM15, SMUG1, TDG, ET2, TET3, TRMT6, TRMT10C, TRMT61B, UHRF1, UNG, WTAP, YTHDC1, YTHDC2, YTHDF1, YTHDF2, YTHDF3, ZBTB4, ZBTB33, ZBTB38, ZC3H13*). (*P* < 0.05)

**Figure S2 Heatmap for the expression levels of 46 regulators within different clinical TNM stages.** Blue type stood for high stage, pink for low stage. The figure showed the expression level of 46 regulatory genes between the two TNM stage groups. TNM 1and 2 stages were defined as low TNM stage (low stage), and TNM 3 and 4 stages as high TNM stage (high stage). 5 regulators expressed significantly between the two subgroups (*ALKBH3, MBD3, RBM15B, UHRF1, ZBTB38*). (**P*<0.05)

**Figure S3 Gene expression treemap of DEGs from the regulators-related risk subgroups.** Each bar represented the different DEG. The size of each bar indicated the specific proportion of average expression in DEG. DEGs: Differentially expression genes.

**Figure S4 Scatter plot of DEGs enriched 37 KEGG pathways.** Y axis: The immune full activation pathway involved DEGs from the regulators-related risk subgroups. X axis: the ratio of the DEGs number to the total gene number in a certain pathway. The color and size of the dots represent the range of the -log10 (*p* value) and the number of DEGs mapped to the indicated pathways, respectively. Cytokine-cytokine receptor pathway was the most enriched pathway.

**Figure S5 Heatmap of 46 regulatory genes in TCGA-SKCM datasets.** Each column represents normalized relative expression of every sample. Each row in the heatmap represents a different regulator.
